# Prevention of Hepatitis B Reactivation in Patients Receiving Immunosuppressive Therapy: a Case Series and Appraisal of Society Guidelines

**DOI:** 10.1007/s11606-022-07806-9

**Published:** 2022-09-22

**Authors:** Samuel Etienne, Jürg Vosbeck, Christine Bernsmeier, Michael Osthoff

**Affiliations:** 1 Division of Internal Medicine, University Hospital Basel, Petersgraben 4, 4031 Basel, Switzerland; 2 Institute of Medical Genetics and Pathology, University Hospital Basel, Basel, Switzerland; 3 University Centre for Gastrointestinal and Liver Diseases, Basel, Switzerland; 4 Department of Clinical Research, University of Basel, Basel, Switzerland

**Keywords:** hepatitis B virus, hepatitis B reactivation, immunosuppression, guidelines, review

## Abstract

Hepatitis B (HBV) reactivation (HBVr) is a potentially fatal complication in patients with past HBV exposure receiving immunosuppressive therapy. HBVr can occur in patients with chronic HBV infection as well as in patients with resolved HBV infection. In this article, we present the cases of four patients with resolved hepatitis B who presented with HBVr during or after immunosuppressive treatment, of whom two died as a consequence of HBVr. We then reflect on and summarize the recommendations of four major societies for the screening and management of previously HBV-exposed patients planned to receive immunosuppressive treatment. Current guidelines recommend screening for HBV in all patients planned to receive immunosuppressive therapy. Risk of HBVr is assessed based on the serological status of the patient and the planned immunosuppressive drug regimen. For patients considered to be at low risk of HBVr, management consists of serological monitoring for HBVr and immediate preemptive antiviral therapy in the case of HBVr. For patients considered to be at intermediate or high risk for HBVr, antiviral prophylaxis should be initiated concordantly with the immunosuppressive therapy and continued for up to 18 months after cessation of the immunosuppressive regimen. Areas of uncertainty include the risk of novel and emerging immunosuppressive and immune modulatory drugs and the exact duration of antiviral prophylaxis. Greater awareness is needed among clinicians regarding the risk of HBVr in patients receiving immunosuppressive therapy, especially in low-endemicity settings. Implementation of screening and management programs and decision support tools based on the presented guidelines may improve the management of these patients.

## INTRODUCTION

Hepatitis B virus (HBV), a member of the Hepadnaviridae family, is an enveloped virus containing partially double-stranded circular DNA. It is the causative agent of an acute or chronic inflammatory disease of the liver.^1^ The virus is transmitted by blood or other body fluids.^2^ The majority of infections worldwide occur following mother-to-child transmission during birth and delivery.^2^ Children have the highest risk to develop chronic hepatitis B (CHB) infection after HBV exposure, reaching 80–90% in infants infected before 1 year of age. Among adults, less than 5% of healthy individuals exposed to HBV develop CHB.^3^

**Figure 1:**
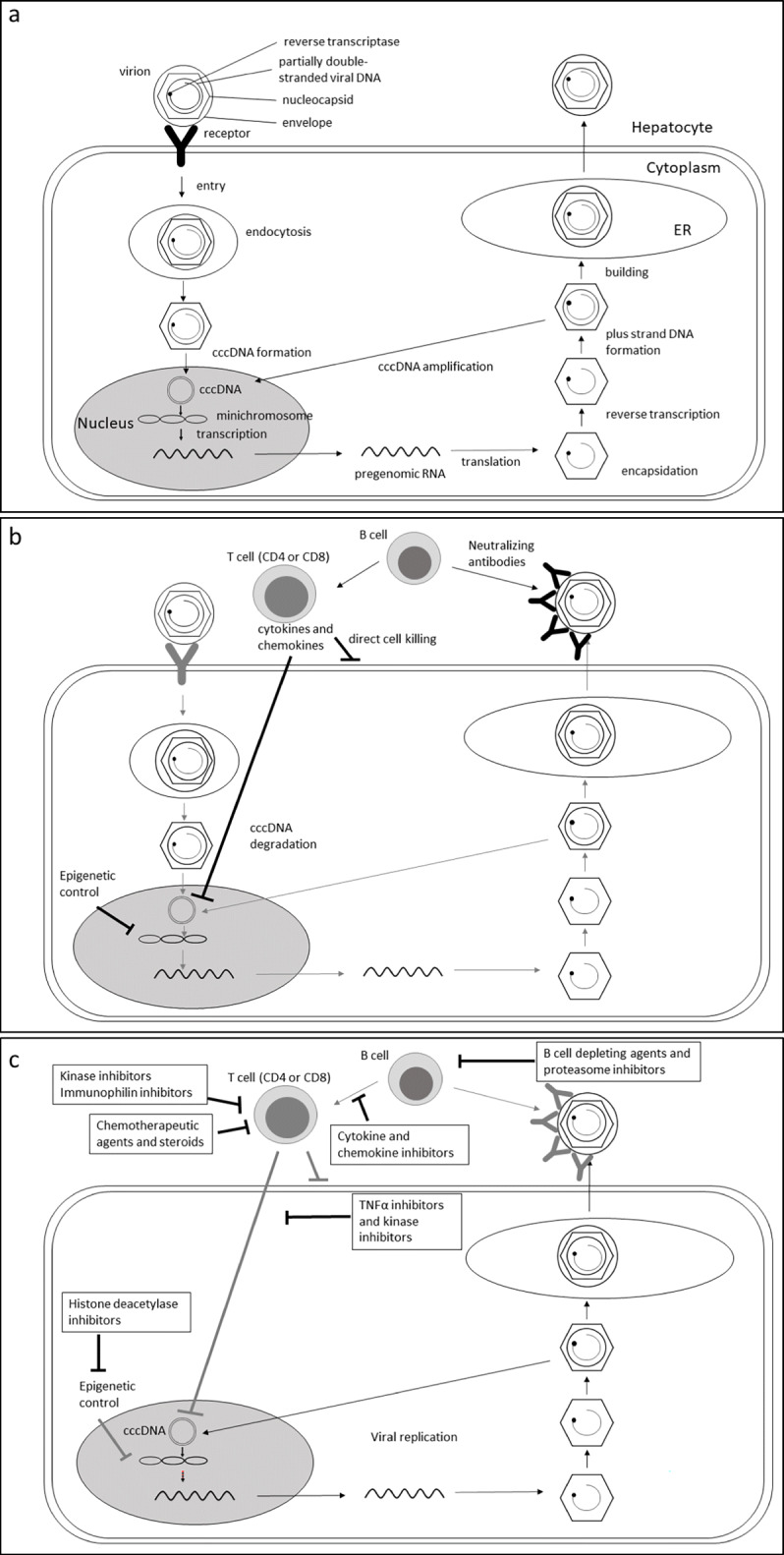
**Schematic representations of hepatitis B virus life cycle (a), immune control of HBV (b), and HBV reactivation in the case of altered or suppressed immune control mechanisms (c). Adapted from [9, 12]. Legend: a Important steps in hepatitis B virus life cycle. b Mechanisms of immune control of HBV. B cells produce neutralizing antibodies and thus prevent viral spread and eliminate circulating viruses. Cytokines can induce a host antiviral pathway causing cccDNA degradation. Histone deacetylase target histone deacetylase, a histone-modifying enzyme important in the epigenetic regulation of gene expression. It has been shown that acetylation of certain histones on the mini-chromosome leads to active gene expression. Conversely, deacetylated histones are associated with transiently silent mini-chromosomes, which is probably the state of HBV genome in individuals with inactive HBV disease. Chemokines trigger the local inflammatory response inducing localization and traffic of activated lymphocytes, important for the local immune control of HBV replication in the liver. Kinase pathways are crucial for immune activation and proliferation of lymphocytes. c Perturbation or suppression of immune control mechanisms by immunosuppressive therapy, leading to hepatitis B virus reactivation. Abbreviations: ER: endoplasmic reticulum; DNA: deoxyribonucleic acid; cccDNA: covalently closed circular DNA; mRNA: messenger ribonucleic acid.**

HBV persists in the body of all infected patients, even those with serological evidence of resolved infection.^4^ After the virion enters the hepatocytes by endocytosis, viral DNA is released into the nucleus, where covalently closed circle DNA (cccDNA) is generated (Fig. 1a). The cccDNA complexes with various host histones, histone-related enzymes, and other proteins to form a mini-chromosome as the template for viral replication,^5^ which is regulated by epigenetic alterations and various transcriptional factors.^6, 7^ The level of cccDNA is augmented and refilled by the replicating HBV DNA via nuclear recycling of nucleocapsid from the cytoplasm.^8^ cccDNA plays an important role in the life cycle of the virus and maintenance of the infection, since it can persist in the nucleus of an infected hepatocyte for the lifetime of the cell,^9^ serving as a reservoir for more cccDNA and new viral particles.^10^

The natural course of infection depends on the interplay between viral replication and the host’s immune system.^4^ HBV-specific T cells either directly target infected cells for elimination via cytopathic mechanisms or suppress viral replication via noncytopathic cytokine-mediated pathways^11^ (Fig. 1b). Neutralizing antibodies produced by activated B cells clear the circulating viruses and further limit the spread of HBV infection. The compelling evidence of B cell suppression leading to HBV reactivation (HBVr) even in resolved hepatitis B underscore that B cells may even exert an additional function in HBV immune control that is not fully understood.^12^ Although these immune control mechanisms are sufficient to control active HBV replication, they are not potent enough to eradicate all of the infected cells containing either latent HBV cccDNA or low-level replicating HBV that escapes the immune control mechanisms. These cells thus constitute a pool of persisting HBV, which is the source of HBVr once the immune control mechanisms are altered or suppressed^12^ (Fig. 1c).

In CHB patients, HBVr is generally defined as an increase in HBV DNA levels in individuals with HBV DNA levels < 2000 IU/ml or reappearance of detectable HBV DNA in individuals without detectable viral DNA. In addition, HBVr also includes the reappearance of detectable HBV DNA or HBsAg in patients with previously resolved HBV (i.e., negative seroconversion).^4, 13^ It can manifest as silent reactivation (elevated viral load without hepatitis), HBV-associated hepatitis (elevated viral load and evidence of clinical, biochemical, or histological hepatitis), or fulminant liver failure (elevated viral load with hepatic dysfunction, coagulopathy, and encephalopathy).^13^ Therefore, HBVr may be associated with significant morbidity and mortality.^14^ Although reactivation may occur spontaneously, immunosuppressive drugs represent the greatest risk of triggering HBVr. This is particularly relevant for anti-CD20 antibodies, such as rituximab (RTX).

Data on HBVr in patients treated with old and novel immunosuppressive therapies are scarce. We present the cases of four patients with resolved hepatitis B who suffered from HBVr during and after a period of immunosuppression and reflect on the guidelines of four major societies regarding the diagnosis and prevention of HBVr in the setting of immunosuppressive treatments.

## CASE PRESENTATIONS

### Patient 1

An 85-year-old man with a history of resolved hepatitis B and diagnosed with a diffuse large B cell lymphoma (DLBCL) was treated with 6 cycles of a RTX-containing chemotherapy regimen [dose-adjusted etoposide, prednisone, vincristine, cyclophosphamide, doxorubicin, and rituximab (EPOCH-R)] and 2 cycles of high-dose methotrexate (MTX) for prophylaxis of a CNS relapse. HBV antiviral prophylaxis was not administered. The patient achieved clinical remissions 8 months after the start of chemotherapy. Three months later, he presented with anorexia and jaundice. HBVr with a significant hepatitis flare was diagnosed (HBV viral load > 170 Mio IU/ml), and tenofovir disoproxil fumarate (TDF) therapy was initiated. Despite therapy, the patient’s condition worsened and he died of liver failure 13 months after initiation of chemotherapy and 2 months after the diagnosis of HBVr.

### Patient 2

**Figure 2:**
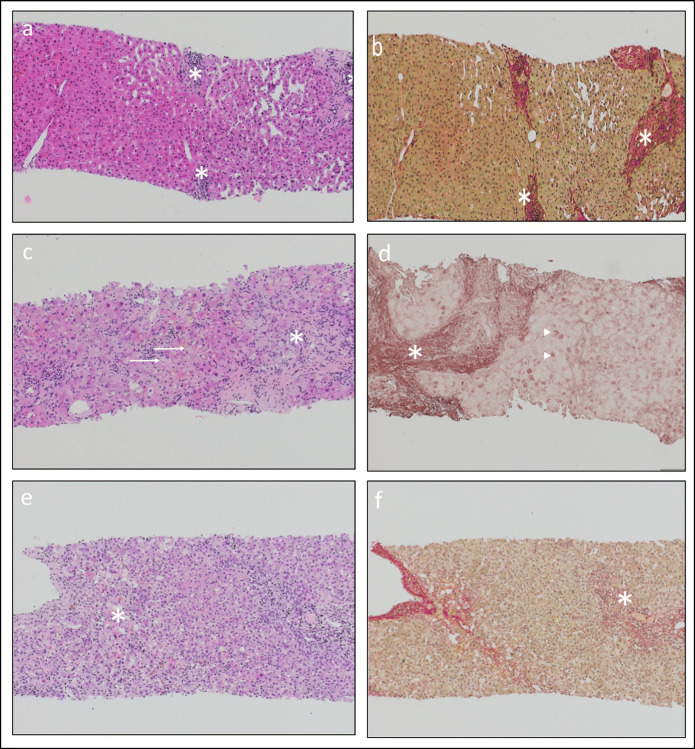
**Liver biopsy of** ***patient 2*** **showing signs of chronic viral hepatitis (a, b): a portal inflammation with little interface activity (asterisks; HE, 100×); b fibrosis (asterisks; Sirius Red, 100×). Liver biopsy of** ***patient 3*** **with signs of acute hepatitis flare (c, d): c portal and lobular inflammation with severe cholestasis (asterisks) and cytoplasmic ground-glass inclusions (long arrows; HE, 100×); d presence of fibrosis (asterisk) and of HBsAg (short arrows; Orcein, 100×). Liver biopsy of** ***patient 4*** **with signs of acute hepatitis (e, f): e portal and lobular inflammation with focally developing necrosis (asterisk; HE, 100×); f absence of relevant fibrosis, formation of so-called passive septa due to confluent necrosis (asterisk; Sirius Red, 100×).**

A 71-year-old man with a history of resolved hepatitis B and treated with mycophenolate mofetil, tacrolimus, and prednisone (7.5 mg/day) since kidney transplantation 8 years ago presented with generalized weakness and poorly controlled glycemia in the setting of a previously well-controlled diabetes mellitus. He was not on HBV antiviral prophylaxis. During workup, elevated liver function test (LFT) results were noticed. HBVr was diagnosed (HBV viral load > 170 Mio IU/ml) and therapy with tenofovir alafenamide (TAF) was established. A significant drop in HBV viral load was observed. Unfortunately, liver biopsy showed evidence of chronic hepatitis with progression of previously known liver fibrosis to cirrhosis (Fig. 2a, b).

### Patient 3

In a 76-year-old man with rheumatoid arthritis, RTX was added to MTX therapy because of insufficient symptom control. Thirteen months later, lamivudine prophylaxis was initiated given serological evidence of resolved hepatitis B. Three months later, he presented with asthenia and abdominal discomfort. HBVr was diagnosed (HBV viral load > 170 Mio IU/ml). Liver biopsy showed signs of acute hepatitis and cirrhosis (Fig. 2c, d). Despite a switch to TAF, the patient developed acute liver failure and died 18 months after the start of the combined immunosuppressive therapy and 5 weeks after the diagnosis of HBVr.

### Patient 4

A 63-year-old woman with a history of resolved hepatitis B and DLBCL presented with jaundice and markedly elevated LFTs. She had achieved clinical remission for DLBCL after RTX-containing chemotherapy [rituximab, cyclophosphamide, doxorubicin, vincristine, and prednisone (R-CHOP)] and 2 cycles of high-dose methotrexate for prophylaxis of a CNS relapse 7 years ago. Antiviral prophylaxis with lamivudine was administered during and until 12 months after cessation of chemotherapy. One week before admission, she had suffered from an upper respiratory tract infection and taken paracetamol up to 3 g per day for symptom control. HBVr was diagnosed (HBV viral load 4 Mio IU/ml,) and therapy with TAF was established. The patient had not received any systemic immunosuppressive therapy during the last 6 years. She only used a combined long-acting beta-agonist and glucocorticoid inhaler for chronic obstructive pulmonary disease (COPD). Liver ultrasound showed normal liver morphology, and the histology of a liver biopsy was consistent with acute hepatitis without fibrosis (Fig. 2e, f). Undetectable HBV viral load and normal LFTs were achieved after 2 months of therapy with TAF.

Laboratory characteristics and histology results of the four patients are presented in Table 1.

**Table 1:** Patients’ Demographic and Clinical Characteristics. All of the Patients Had Serological Evidence of Resolved Hepatitis B (HBsAg−/Anti-HBc+) and Liver Function Tests Within the Normal Range Before Initiation of Immunosuppressive Therapy

Patient	Indication for IST and regimen	PPX	HBV reactivation (HBVr)				Outcome	HBVr caused by IST
			*Timing (since IST start)*	*Serology, HBV DNA levels (Mio IU/l), LFTs (U/l), and bilirubin (μmol/l)*	*Clinical presentation, ultrasound and histology*	*Therapy*		
1 (m, 85 y)	Lymphoma (DLBCL) RTX-based chemotherapy (EPOCH-R)	No	11 months	HBsAg+, HBeAg+ DNA > 170 AST 314 ALT 275 GGT 408 Bilirubin 82	Jaundice, ascites. Ultrasound: normal liver morphology. Liver biopsy: not performed	TDF	Liver failure, death	Yes
2 (m, 71 y)	Kidney transplantation MMF, MTX, prednisone 7.5 mg/d	No	8 years	HBsAg+, HBeAg+ DNA > 170 AST 42 ALT 31 GGT 157 Bilirubin 23	Ascites. Elastography: progression of previously known liver fibrosis. Liver biopsy: chronic hepatitis, cirrhosis	TAF	Alive with cirrhosis	Possibly
3 (m, 76 y)	Rheumatoid arthritis MTX and RTX	No (lamivudine only started after 13 months)	16 months	HBsAg-, HBeAg- DNA >170 AST 319 ALT 311 GGT 332 Bilirubin 49	Ascites. Ultrasound: normal liver morphology. Liver biopsy: acute hepatitis, cirrhosis	TDF	Liver failure, death	Yes
4 (f, 63 y)	Lymphoma (DLBCL) RTX-based chemotherapy (R-CHOP)	Yes: lamivudine until 12 months after IST cessation	7 years	HBsAg+, HBeAg+ DNA 4 AST 1491 ALT 1233 GGT 438 Bilirubin 386	Jaundice. Ultrasound: normal liver morphology. Liver biopsy: acute hepatitis, no fibrosis	TAF	Viral load not detectable and resolution of hepatitis.	Probably not

*Abbreviations*: *m* male, *f* female, *y* years, *IST* immunosuppressive therapy, *HBV* hepatitis B virus, *PPX* antiviral prophylaxis, *LFTs* liver function tests, *DLBCL* diffuse large B cell lymphoma, *EPOCH-R* etoposide, prednisone, vincristine, cyclophosphamide, doxorubicin, and rituximab, *RTX* rituximab, *MMF* mycophenolate mofetil, *MTX* methotrexate, *R-CHOP* rituximab, cyclophosphamide, doxorubicin, vincristine, and prednisone, *HbsAg* hepatitis b surface antigen, *HBeAg* hepatitis B e antigen, *ALT* alanine aminotransferase, *AST* aspartate aminotransferase, *TDF* tenofovir disoproxil fumarate, *TAF* tenofovir alafenamide, *HBVr* hepatitis B virus reactivation

## HEPATITIS B INFECTION

In 2019, the World Health Organization estimated the number of individuals with CHB at 296 million worldwide, with 1.5 million new infections every year.^2^ High-endemicity areas are Asia, Africa, and the Amazon River basin. Eastern Europe, the Mediterranean, and Central and South America are intermediate-prevalence regions. The prevalence is low in North America, Australia, and large parts of Europe including Switzerland.^15^

Serology is essential to diagnose both chronic HBV and resolved HBV infection. Detection of positive HBV core antibodies (anti-HBc) is consistent with prior exposure to HBV. Patients with chronic HBV infection (CHB) usually have serological evidence of HBV surface antigen (HBsAg+). Resolved HBV infection is diagnosed in the absence of HBsAg and of detectable HBV DNA levels in the blood.^14^

HBVr may occur in the setting of resolved and chronic HBV infection and is defined as an increase or reappearance of HBV DNA. Risk factors for HBVr are classified in three categories: (1) host factors, namely male sex, older age, presence of liver cirrhosis, and disease needing immunosuppressive therapy (IST); (2) virological factors, i.e., high-baseline HBV DNA level, hepatitis B e antigen (HBe Ag) positivity, CHB, and coinfection with the hepatitis C virus, hepatitis D virus, or human immunodeficiency virus; and (3) nature and degree of immunosuppression.^12, 16, 17^

## HEPATITIS B REACTIVATION IN THE SETTING OF IMMUNOSUPPRESSIVE THERAPY

Reactivation can occur spontaneously (as in case 4), but it is more frequently observed during immunosuppressive therapy (as in cases 1–3). Immunosuppressive drugs represent the greatest risk of triggering HBVr.^13^ In the setting of chronic immunosuppressive therapy, both patients with CHB and patients with resolved HBV infection are at risk for HBVr.^14^ Between 17 and 55% of patients with previous HBV exposure experience HBVr if not prescribed antiviral prophylaxis in this setting.^18^

Immunosuppressive drugs have been categorized according to their potential to cause HBVr.^19, 20^ B cell–depleting agents are among the drugs with the highest risk (anticipated incidence of HBVr > 10%).^13^ B cells produce neutralizing antibodies that eliminate circulating viruses, and thus, they play a key role in the humoral immune response, contributing to the control of HBV. B cell–depleting agents, such as RTX, ocrelizumab, and ofatumumab, are monoclonal antibodies binding to CD20, a cell surface marker on B lymphocytes, thus killing B lymphocytes via cytotoxicity and apoptosis.^21^ These B cell–depleting agents are therefore associated with a particularly high risk of HBVr.^22^

## SOCIETY RECOMMENDATIONS FOR THE MANAGEMENT OF PATIENTS AT RISK OF HBVR

HBVr is preventable. Identifying patients at risk and initiating adequate treatment or close surveillance is important because a delayed detection of HBVr or the failure to start prophylaxis may lead to severe hepatic injury and liver failure that may not be reversible, even after the initiation of antiviral therapy (such as in cases 1–3).^22^ Several major international gastroenterology and hepatology organizations have published recommendations for the testing and management of HBV infection in patients receiving immunosuppressive therapy: the American Gastroenterological Association (AGA),^19^ the European Association for the Study of the Liver (EASL),^23^ the American Association for the Study of Liver Diseases (AASLD),^24^ and the Asian Pacific Association for the Study of the Liver (APASL).^20^ Small differences aside, the guidelines agree in the general principles for the prevention of HBVr which include (1) recognizing the need to screen patients prior to the initiation of immunosuppression, (2) stratifying the risk of HBVr based on serostatus and immunosuppressive regimen, and (3) tailoring management strategies based on the risk and including close monitoring with preemptive treatment or antiviral prophylaxis.

### Screening

Most guidelines recommend screening for HBV infection in all patients planned to receive immunosuppressive regimens including HBsAg and anti-HBc, followed by a HBV DNA test if either one is positive. The role of anti-HBs screening in addition to HBsAg and anti-HBc is controversial: EASL and APASL recommend anti-HBs as a part of screening, while AGA and AASLD do not. The role of anti-HBs screening is even less clear, since the presence of anti-HBs does not prevent HBVr. However, the level of anti-HBs may provide additional information regarding the risk of HBVr, as a titer > 100 IU/ml has been associated with a decreased HBVr incidence.^25^ In addition, the presence of anti-HBs may confirm past infection in HBsAg−/anti-HBc+ patients, and conversely, a loss of anti-HBs may be a predictor of HBVr.^24^ APASL furthermore recommends the assessment of the degree of liver fibrosis in all CHB patients and in all patients with resolved HBV infection, since patients with advanced fibrosis and cirrhosis are at higher risk of mortality and morbidity in case of HBVr.

### Risk Stratification

After screening, the next step is risk stratification based on serological status and immunosuppressive regimen. In general, CHB patients have a higher risk for HBVr than patients with resolved HBV (e.g., HBVr risk of > 30% vs. 10% after rituximab treatment).^19, 20, 23, 24^ AASLD mentions the difficulty of determining the specific risk of a particular drug or drug regimen because of a lack of systematically collected data and the exclusion of CHB or even resolved HBV patients in clinical trials.^26–29^ AASLD and EASL recommend starting antiviral prophylaxis in all CHB patients planned to receive immunosuppressive therapy and in patients with resolved HBV infection planned to receive B cell–depleting agents or undergoing stem cell transplantation. For patients with resolved HBV infection planned to receive any other immunosuppressive therapy, monitoring with preemptive treatment rather than antiviral prophylaxis may be considered. In 2021, APASL has issued a classification based on serological status and the type and duration of immunosuppressive therapy (Table 2),^20^ which is in line with a previously published classification by AGA.^19^ The risk is defined as low if the incidence of HBVr is < 1%, intermediate if HBVr incidence is between 1 and 10%, and high if the incidence of HBVr is > 10%.^19, 20^ The remarkably high risk of B cell–depleting therapies and hematopoietic stem cell transplantation (HSCT) for both CHB and resolved hepatitis B patients is emphasized in all guidelines.

**Table 2:** Definition of Immunosuppressive Therapy Risk Categories According to APASL

	Low risk (< 1%)	Moderate risk (1–10%)	High risk (> 10%)	Uncertain
CHB	Methotrexate Azathioprine 6-Mercaptopurine Low-dose CS DAA for HBV/HCV coinfection with HBsAg < 10 IU/ml	Cytotoxic chemotherapy (excluding anthracycline derivates) TNF-α inhibitors with lower potency* Moderate-dose CS Proteasome inhibitor	B cell–depleting therapy High-dose CS TNF-α inhibitors with higher potency^†^ Anthracycline derivates HSCT (autologous and allogenic) Immune checkpoint inhibitors*:* *Anti-PD-1, anti-PD-L1, anti-CTLA-4* Tyrosine kinase inhibitors	Abatacept, Tocilizumab, Ibrutinib, Alemtuzumab, Natalizumab, Ocrelizumab, Ibritumomab
Resolved HBV infection	Cytotoxic chemotherapy (excluding anthracycline derivates) High-dose CS TNF-α inhibitors with lower potency* DAA for HCV	Anthracycline derivates Autologous HSCT TNF-α inhibitors with higher potency^†^	B cell–depleting therapy Allogenic HSCT	Immune checkpoint inhibitors: *anti-PD-1 niolumab, pembrolizumab, anti-PD-L1: atezolizumab, anti-CTLA-4: ipilimumab*

Abbreviations: *CHB* chronic hepatitis B infection; *HBV* hepatitis B virus; *CS* corticosteroids; *low-dose CS* < 10 mg/day prednisone or equivalent for ≥ 4 weeks; *moderate-dose CS* 10–20 mg/day prednisone or equivalent for ≥ 4 weeks; *high-dose CS* ≥ 20 mg/day prednisone or equivalent for ≥ 4 weeks; *TNF-α inhibitors* tumor necrosing factor alpha inhibitors: etanercept, adalimumab, certolizumab, infliximab; *HSCT* hematopoietic stem cell transplant

*TNF-α inhibitors with lower potency: etanercept

^†^TNF-α inhibitors with higher potency: adalizumab, infliximab, golimumab, certolizumab; anthracycline derivates: doxorubicin, epirubicin

### Management

**Figure 3:**
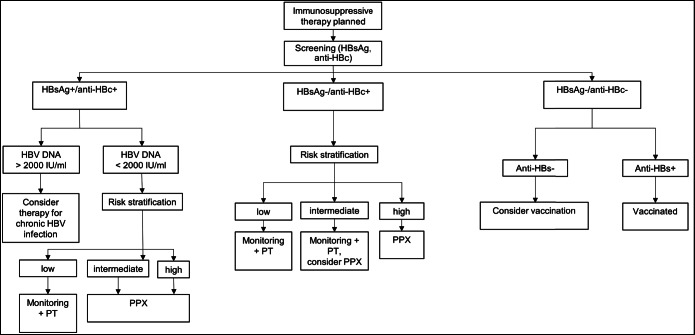
**Proposed algorithm for the management of patients with CHB or resolved hepatitis B infection planned to receive IST based on current society guidelines, adapted from.**^20, 22^ ***Abbreviations*: HBsAg: hepatitis B surface antigen; anti-HBc: hepatitis B core antibody; +: positive; -: negative; risk stratification: AGA**^19^ **and APASL**^20^ **have issued classifications based on serological status and the type and duration of immunosuppressive therapy, risk is defined as low if the incidence of HBVr is < 1%, intermediate if HBVr incidence is between 1 and 10%, and high if the risk is > 10%; PT: preemptive therapy; monitoring + PR consists of monitoring of liver function tests, HBV, DNA, and HBsAg every 1–3 months and start of antiviral therapy if elevated HBV DNA or a positive HBsAg is detected; PPX: antiviral prophylaxis with nucleos(t)ide analogues.**

The prevention strategy, consisting of monitoring with preemptive therapy or antiviral prophylaxis, is tailored according to HBV disease status and immunosuppressive regimen (Fig. 3). Monitoring with preemptive therapy is recommended either for CHB and treatment with drugs involving a low reactivation risk or for resolved HBV infection patients and treatment with a low or intermediate risk of reactivation drug regimen. In contrast, antiviral prophylaxis is indicated for CHB patients receiving a drug regimen with an intermediate or high risk of HBVr or resolved HBV infection patients with a high-risk HBVr treatment.

Monitoring strategies consist of LFTs, HBsAg, and HBV DNA measurements every 1–3 months and immediate start of nucleos(t)ide analogue (NA) therapy in the case of HBVr.

For patients in whom antiviral prophylaxis is recommended, antiviral therapy with NA should ideally be started 1 week before the start of immunosuppressive therapy. EASL recommends monitoring of liver function tests (LFTs) and HBV DNA every 3–6 months in patients on antiviral prophylaxis (Table 3). NA treatment is recommended for 6–12 months after stopping immunosuppressive treatment, with longer (12–18 months) antiviral prophylaxis durations recommended in the case of B cell–depleting agents. EASL and AASLD recommend continuing the monitoring 12 months after termination of NA therapy. Both for antiviral prophylaxis and treatment, NAs with a high barrier to resistance such as entecavir, TAF, or TDF are recommended. EASL recommends lamivudine or NAs with a high barrier to resistance in patients with resolved hepatitis B receiving high-risk immunosuppressive treatment. TDF/TAF and entecavir are less likely to induce drug-resistant virus and are more likely to achieve viral suppression compared with lamivudine, in particular when long-term treatment is required.

**Table 3:** Recommendations for the Management of HBV-Infected Patients Due to Receive Immunosuppressive Regimens According to Society Guidelines

Society	Strategy	NA regimen	Duration of NA	Monitoring during and after IST	Follow-up
AGA (2015)	**CHB** High risk: PPX Moderate risk: PPX, may consider monitoring and on-demand therapy (PT strategy) Low risk: monitoring with PT **Resolved HBV infection** High risk: PPX Moderate: PPX, can consider monitoring with PT Low risk: monitoring with PT	Antivirals with high barrier to resistance	≥ 6 months after IST termination, ≥ 12 months if on B cell–depleting therapy	–	–
EASL (2017)	**CHB** PPX **Resolved HBV infection** B cell–depleting therapy for oncological indication, HSCT: PPX Other therapies: monitoring with PT, PPX recommended in case of long duration of IS, limited compliance to monitoring, unknown risk in new biologicals	**CHB**: ETV, TAF, TDF **Resolved HBV infection**: LAM, ETV, TAF, TDF	≥ 12 months after IST termination, ≥ 18 months if on B cell–depleting therapy or HSCT	PPX: monitor LFTs and HBV DNA every 3–6 months Monitoring with PT: monitor HBsAg and/or HBV DNA every 1–3 months during and after IST, and start NA if HBV DNA+ and/or HBsAg+	≥ 12 months after NA termination: LFTs and HBV DNA every 3–6 months
AASLD (2018)	**CHB** PPX **Resolved HBV infection** B cell–depleting therapy, HSCT: PPX Other therapies: PPX, can consider monitoring with PT	ETV, TAF, TDF	≥ 6 months after IST termination, ≥ 12 months if on B cell–depleting therapy	Monitoring with PT: monitor ALAT, HBV DNA, and HBsAg every 1–3 months, start NA if HBV DNA+ and/or HBsAg+	Continue up to12 months after NA termination, especially in the case of B cell–depleting therapy
APASL (2021)	**CHB** High and moderate risk: PPX Low risk with or without advanced fibrosis or cirrhosis: PPX Low risk without advanced fibrosis or cirrhosis: monitoring with PT **Resolved HBV infection** High-risk group: PPX Moderate and low risk with advanced liver fibrosis or cirrhosis: PPX. Low and moderate risk without advanced fibrosis or cirrhosis: monitoring with PT	ETV, TAF, TDF	6 months after IST termination, consider termination of NA and start monitoring for: HBsAg+ patients without advanced liver fibrosis or cirrhosis, for HBsAg+ patients with low levels of HBV DNA (< 2000 IU/ml) before initiation of N, resolved hepatitis B patients	Monitoring with PT: monitor ALT, AST, bilirubin, albumin every 3 months, if ALT > 2× baseline, check HBsAg and HBV DNA, start NA if HBV DNA+ and/or HBsAg+	-

*Abbreviations*: *AGA* American Gastroenterology Association, *EASL* European Association for the Study of the Liver, *AASLD* American Association for the Study of Liver Disease, *APASL* Asia-Pacific Association for the Study of the Liver, *IST* immunosuppressive therapy, *CHB* chronic hepatitis B infection, *HBV* hepatitis B virus, *ALT* alanine aminotransferase, *AST* aspartate aminotransferase, *DNA* deoxyribonucleic acid, *HBV* hepatitis B virus, *HBsAg* HBV surface antigen, *HSCT* hematopoietic stem cell transplant, *IST* immunosuppressive therapy, *NA* nucleos(t)ide analogue, *PPX* antiviral prophylaxis, *PT* preemptive treatment, *RTX* rituximab, *LFTs* liver function tests, *LAM* lamivudine, *ETV* entecavir, *TAF* tenofovir alafenamide, *TDF* tenofovir disoproxil fumarate

### Specific Considerations for Renal Transplantation

EASL and AASLD have issued specific recommendations for non-liver solid organ transplant recipients including screening in all patients listed for solid organ transplantation. In HBsAg+ patients, ASSLD recommends further staging of liver disease with liver biopsy and elastography. Long-term antiviral prophylaxis for CHB patients and monitoring with preemptive therapy for those with resolved HBV infection are recommended by both societies. In the AASLD guidelines, a monitoring period of 1 year after transplantation and after treatment with T cell–depleting agents is discussed.

## DISCUSSION

HBVr remains an important cause of morbidity and mortality among patients with CHB and resolved HBV infection receiving immunosuppressive therapy if adequate management with monitoring and preemptive therapy or antiviral prophylaxis is not considered beforehand. There is broad consensus among major societies on the management of these patients.

The four patients presented had serologies compatible with resolved HBV infection. According to current guidelines, antiviral prophylaxis is recommended in patients 1 and 3, who were treated with RTX, a B cell–depleting agent considered to have a high risk of HBVr. In patient 2, the presence of elevated LFTs prompted HBV DNA measurement and eventually the start of NA therapy. In the setting of renal transplantation, preemptive therapy with monitoring of LFTs, HBV DNA, and HBsAg may allowed earlier recognition of HBVr. Accordingly, earlier start of NA therapy might have prevented the progression of liver disease, although the monitoring period is not clear.

Patient 4 received antiviral prophylaxis with lamivudine during and for 12 months after cessation of RTX. In the absence of antiviral prophylaxis, HBVr has been reported to occur as late as 12 months (rarely even later^30^) after cessation of immunosuppressive treatment both in CHB and resolved HBV infection patients.^31^ Presumably, HBVr in patient 4 was not triggered by RTX 6 years after its cessation. However, the required duration of antiviral prophylaxis has not yet been studied in randomized controlled trials.^12^ The patient used inhaled corticosteroids for COPD. A retrospective study reported HBVr rates of 3.1% in CHB patients with asthma or COPD treated with inhaled corticosteroids.^32^ To our knowledge, there are no available data regarding the rate of HBVr during therapy with inhaled corticosteroids in patients with resolved HBV infection. Apart from her age, no other risk factors for HBVr were evident, and a spontaneous HBVr is possible. Therefore, a high index of suspicion for HBVr is warranted in the case of LFT abnormality in patients with resolved HBV infection.

The lack of monitoring and antiviral prophylaxis in two of the presented patients, respectively, might reflect a lack of awareness of the risk of HBVr when prescribing immunosuppressive drugs in patients with resolved HBV infection. Particularly in low-endemicity regions such as Switzerland, physicians are not frequently confronted with patients with previous HBV exposure or chronic HBV infection.

Insufficient HBV screening rates and low rates of antiviral therapy have been observed in other recent studies.^33–36^ The reasons elaborated on by the authors are mainly a lack of awareness among clinicians regarding the risk of HBVr, non-adherence to existing guidelines, lack of knowledge about appropriate management strategies, and misinterpretation of HBV serological results. Implementation of institutional quality-improvement measures such as pre-prescribing workflows, order sets, checklists, and clinical decision support has been shown to be effective in increasing the rates of correctly identified and adequately managed at-risk patients.^37, 38^ Thus, further efforts regarding education of clinicians and institutional initiatives for quality improvement are warranted.

At our institution, we are currently implementing an automatic alert system for clinicians in case of the prescription of high-risk immunosuppressive treatments such as rituximab. The alert informs about the necessity of HBV screening and, in the case of positive HBsAg and/or anti-HBc results, about the requirement of reviewing the indication for antiviral prophylaxis or monitoring with preemptive therapy.

Some questions remain unanswered after the review of current society guidelines. The discrepancies in the risk estimation of some drugs in the AGA and APASL guidelines highlight the uncertainty in the risk assessment. The lack of systematically collected data is a major obstacle to determine the HBVr risk for many drugs. This limitation is underscored by the fact that immunosuppressive drugs are often given in combinations. Further studies and pharmacovigilance programs are needed to assess the risks of HBVr caused by novel biological therapies. This is highlighted in a recent study, which reports HBVr in 6.5% of patients with resolved HBV infection after therapy with daratumumab for multiple myeloma. Daratumumab is a human immunoglobin G1 monoclonal antibody targeting CD38-expressing cells. It causes death of CD38-expressing myeloma cells, but it also targets CD38-expressing plasma cells, which might cause the loss of protective immunity against viral infections such as HBV.^39^ Immune checkpoint inhibitors; Bruton tyrosine kinase (BTK) inhibitors; agents targeting CD22, CD38, and CCR4; and chimeric antigen receptor T cell therapy have also been associated with HBVr, although HBVr mostly occurred in patients with CHB and only occasionally in patients with resolved HBV infection.^40^ Until more evidence is available about the exact risk profiles of these agents, adopting strategies including antiviral prophylaxis or monitoring with preemptive therapy similar to those recommended for high-risk immunosuppressive agents seems reasonable.

In summary, we suggest the following recommendations for everyday clinical practice. Screening is warranted for every patient planned to receive immunosuppressive therapy. The management strategy is then adopted according to the patient’s serological status and the planned drug regimen. Due to their favorable side effect profile, we favor antiviral prophylaxis if in doubt regarding the risk of HBVr or if close monitoring is not feasible. We have provided an algorithm for busy clinicians who seek an overview of the recommended management strategies (Fig. 3). To clinicians seeking in-depth society recommendations for daily practice, we suggest the AGA 2015 guidelines^19^ for their conciseness, the included risk classification of immunosuppressive therapies, and the clinical decision support tool provided.^41^ The other guidelines presented in this article furthermore address the duration of prophylaxis or monitoring depending on the immunosuppressive regimen and HBVr risk.

## CONCLUSION

We present a case series of four patients with HBVr in the setting of resolved HBV and present or past immunosuppressive therapy illustrating that HBVr is a potentially fatal, yet preventable, complication. Current society guidelines provide important guidance on screening and management strategies. Quality-improvement strategies and education of clinicians prescribing potent immunosuppressive drugs to increase awareness of the risk of HBVr in patients with CHB or resolved HBV infection are of paramount importance.

## Abbreviations

AASLD: American Association for the Study of Liver Diseases
AGA: American Gastroenterological Association
Anti-HBc: Hepatitis B core antibody
APASL: Asian Pacific Association for the Study of the Liver
ALT: Alanine aminotransferase
AST: Aspartate aminotransferase
cccDNA: Covalently closed circular DNA
CHB: Chronic hepatitis B
CS: Corticosteroids
DNA: Deoxyribonucleic acid
EASL: European Association for the Study of the Liver
ETV: Entecavir
HBV: Hepatitis B virus
HBVr: HBV reactivation
HBsAg: HBV surface antigen
HSCT: Hematopoietic stem cell transplant
LFTs: Liver function tests
MTX: Methotrexate
NA: Nucleos(t)ide analogue
PPX: Antiviral prophylaxis
RTX: Rituximab
TAF: Tenofovir alafenamide
TDF: Tenofovir disoproxil fumarate
